# Antimalarial activity of cecropin antimicrobial peptides derived from *Anopheles* mosquitoes

**DOI:** 10.1128/aac.00311-24

**Published:** 2024-06-14

**Authors:** Junchao Lou, Dongying Zhang, Jingyao Wu, Guoding Zhu, Meihua Zhang, Jianxia Tang, Yaqun Fang, Xiaoqin He, Jun Cao

**Affiliations:** 1 School of Public Health, Nanjing Medical University, Nanjing, Jiangsu, China; 2 National Health Commission Key Laboratory of Parasitic Disease Control and Prevention, Jiangsu Provincial Key Laboratory on Parasite and Vector Control Technology, Jiangsu Institute of Parasitic Diseases, Wuxi, China; 3 Kunming Institute of Zoology, Chinese Academy of Sciences, Kunming, Yunnan, China; The Children's Hospital of Philadelphia, Philadelphia, Pennsylvania, USA

**Keywords:** malaria, drug resistant, *Anopheles*, antimicrobial peptides, antimalarial peptides

## Abstract

The emergence of clinically drug-resistant malaria parasites requires the urgent development of new drugs. Mosquitoes are vectors of multiple pathogens and have developed resistance mechanisms against them, which often involve antimicrobial peptides (AMPs). An-cecB is an AMP of the malaria-transmitting mosquito genus *Anopheles*, and we herein report its antimalarial activity against *Plasmodium falciparum* 3D7, the artemisinin-resistant strain 803, and the chloroquine-resistant strain Dd2 *in vitro*. We also demonstrate its anti-parasite activity *in vivo*, using the rodent malaria parasite *Plasmodium berghei* (ANKA). We show that An-cecB displays potent antimalarial activity and that its mechanism of action may occur through direct killing of the parasite or through interaction with infected red blood cell membranes. Unfortunately, An-cecB was found to be cytotoxic to mammalian cells and had poor antimalarial activity *in vivo*. However, its truncated peptide An-cecB-1 retained most of its antimalarial activity and avoided its cytotoxicity *in vitro*. An-cecB-1 also showed better antimalarial activity *in vivo*. Mosquito-derived AMPs may provide new ideas for the development of antimalarial drugs against drug-resistant parasites, and An-cecB has potential use as a template for antimalarial peptides.

## INTRODUCTION

Malaria parasites are transmitted to mammalian host *via* the bites of infected *Anopheles* mosquitoes. There were an estimated 249 million malaria cases in 2022 (1), the majority of which were of *Plasmodium falciparum* and occurred in Africa (2). There are two vaccines currently available for malaria [RTS,S/ASO1 (Mosquirix) and R21/Matrix-M (R21/MM)], but their relatively low to modest efficacy lessens their potential as eradication tools (3, 4), and their introduction is unlikely to reduce the demand for antimalarial drugs.

At present, the antimalarial drugs used for clinical treatment are derived from three drug classes: artemisinins, aminoquinolines, and antifolates, and *Plasmodium falciparum* has evolved resistance to each of these (5). Artemisinin-based combination therapies (ACTs) have been officially recommended as a first-line drug by the WHO since 2006 (6). Unfortunately, the sensitivity of *P. falciparum* to artemisinin has declined in the Greater Mekong Subregion, and the emergence of multi-drug resistant strains has been observed in South America and Southeast Asia (7). Artemisinin-resistant parasites have been recorded from the entire Greater Mekong River Basin area, including Cambodia, Laos, Myanmar, Thailand, Vietnam and other countries (8, 9). The development of new antimalarial drugs with distinct modes of action is urgently needed.

It has been observed that particular *Plasmodium* strains are unable to develop in certain refractory mosquito strains due to innate immune responses, especially some key components of the mosquito complement-like system (10–13). Even in fully susceptible mosquito strains, large numbers of parasites are lost during sporogonic development in the mosquito (11–13). These losses are correlated with the transcriptional activation of mosquito immune genes (14–16). Antimicrobial peptides (AMPs), key factors in fighting infections in insects, are inextricably linked to the body’s complement system and may be involved in malaria parasite elimination (16–21). AMPs are small-molecule active peptides produced in a variety of organisms and are important parts of natural immune defense systems against exogenous pathogenic microorganisms (20–22). In addition to immune defense, many peptides have been demonstrated to have direct activity against *Plasmodium* parasites in both mosquitoes and vertebrates (23–25). AMPs are less likely to induce drug resistance in pathogenic microorganisms than conventional drugs, as their main mode of action is to form pores in membranes (22, 23, 26). Mosquito-derived AMPs may offer a potent source of potential antimalarial compounds.

The repertoire of mosquito AMPs includes defensins, gambicins, cecropins, attacin, diptericin, and holotricin (24, 26–28). Of them, diptericin and holotricin are glycine-rich peptides with about 8 kDa in mass and thought to have restricted activity toward Gram-negative bacteria (27). Although attacins have been shown to be active against a range of microbes including Gram-positive and Gram-negative bacteria, fungi, and protozoal parasites, compared with several other antimicrobial peptides, they have a larger mass of 23 kDa (28). Defensins possess six conserved cysteine residues forming three intramolecular disulfide bonds, and gambicins have eight cysteine residues forming four intramolecular disulfide bonds. Cecropins are linear peptides lacking cysteine residues with a low molecular weight of about 4 kDa (29). As the presence of multiple disulfide bonds can complicate synthesis and increase cost of production, especially in the case of multiple disulfide bonds, cecropins are cheaper to synthesize compared with defensins and gambicins. There are currently few studies on the antimalarial activities of mosquito-derived cecropins. There are three main cecropin genes A, B, and C in *Anopheles* (30). In this study, one peptide from each of the three cecropin genes of *Anopheles arabiensis* (A, B, and C) was synthesized and their antimalarial activities were analyzed.

## RESULTS

### Sequence and physicochemical properties of the peptides

The sequences of all the studied peptides are from *Anopheles arabiensis*, and their physicochemical properties, including molecular weight (MW), isoelectric point (PI), and grand average of hydropathicity (GRAVY), analyzed using the ProtParam tool available on the ExPASy website (Expasy - ProtParam tool) are shown in Table 1. Their PI ranged from 10.47 to 12.02. In addition to the PI values, their hydrophilicity and hydrophobicity were analyzed using GRAVY values. Hydrophilic peptides have GRAVY values lower than 0 and hydrophobic peptides values greater than 0. GRAVY score values of An-cecA, An-cecB, An-cecC, and the truncated peptides An-cecB-1 and An-cecB-3 were more hydrophilic with GRAVY values between −1.100 and −0.021. However, the truncated peptide An-cecB-2 was hydrophobic with a GRAVY value of 0.362. All peptides were cationic peptides with positive charges.

**TABLE 1:** Sequences and physicochemical properties of peptides^*a*^

Name	Gene ID	Sequence	Net charge	MW (Da)	PI	GRAVY
An-cecA	XP_040173531	GRLKKLGKKIEGAGKRVFKAA EKALPVVAGVKAL-NH2	+8	3,783.61	10.79	−0.021
An-cecB	XP_040173530	APRWKFGKRLEKLGRNVFRAA KKALPVIAGYKAL-NH2	+9	4,105.94	11.61	−0.309
An-cecC	XP_040172706	RRFKKFLKKVEGAGRRVANAA QKGLPLAAGVKGL-NH2	+9	3,887.64	12.02	−0.332
An-cecB-1	–	APRWKFGKRLEKLGRNVF-NH2	+5	2,453.88	11.73	−0.906
An-cecB-2	–	RAAKKALPVIAGYKAL-NH2	+4	1,921.32	10.46	0.362
An-cecB-3	–	GKRLEKLGRNVFRAAKK-NH2	+6	2,222.63	11.74	−1.100

^*a*^ MW, molecular weight; PI, isoelectric point; GRAVY, grand average of hydropathicity; –, derived peptide, no Gene ID.

### Preliminary screening of the *in vitro* antimalarial activity of AMPs

**Fig 1:**
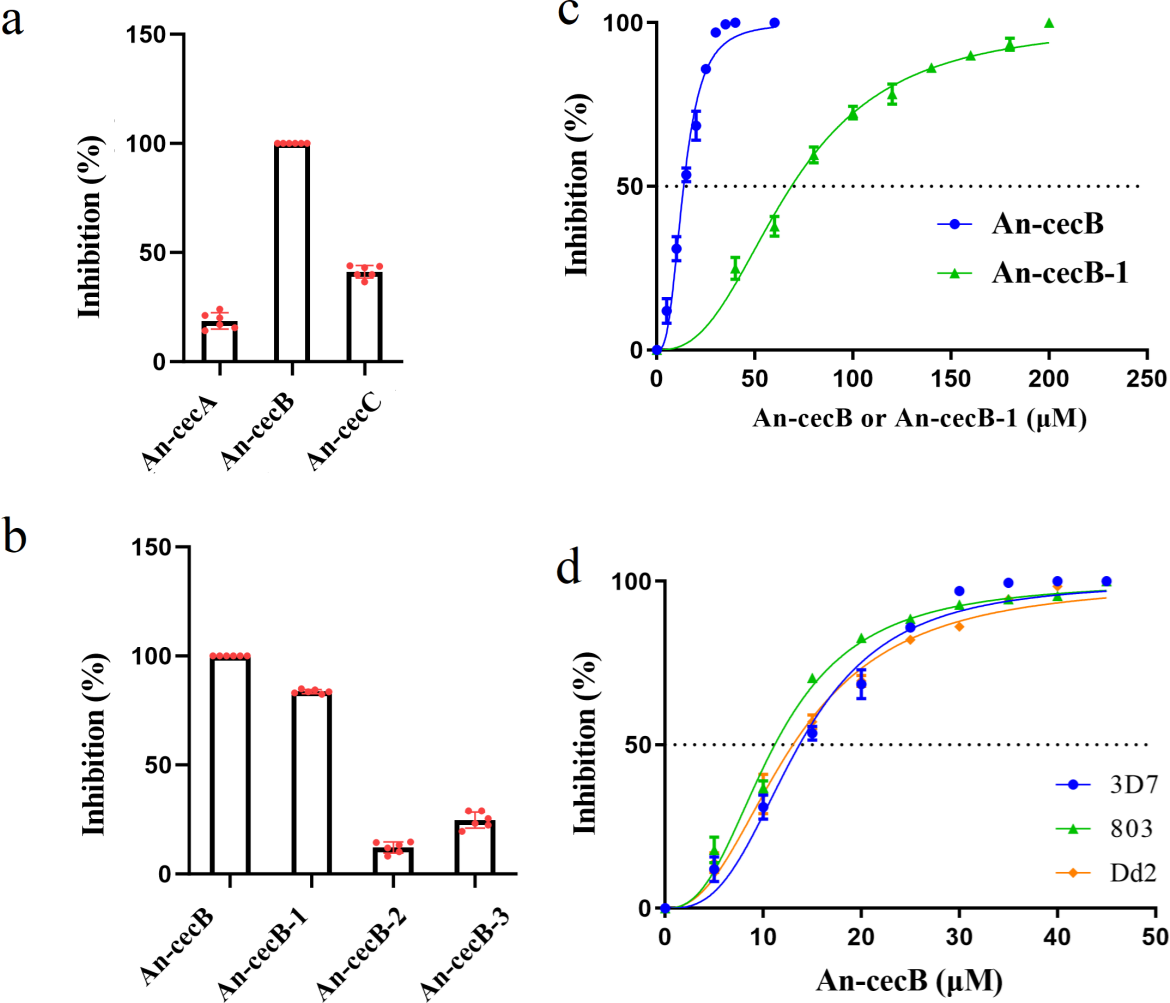
Preliminary screening of *in vitro* antimalarial activity of antimicrobial peptides. (**a**) *In vitro* antimalarial activity of An-cecA, An-cecB, and An-cecC at a concentration of 50 µM against *P. falciparum* 3D7. (**b**) *In vitro* antimalarial activity of An-cecB at 50 µM and its truncated peptides An-cecB-1, An-cecB-2, and An-cecB-3 at 200 µM against *P. falciparum* 3D7. (**c**) IC50s of An-cecB and its truncated peptide An-cecB-1 against *P. falciparum* 3D7. (**d**) IC50s of An-cecB against *P. falciparum*-sensitive strain 3D7, artemisinin-resistant strain 803, and chloroquine-resistant strain Dd2. Data are individual replicates or means ± SEM of 10,000 red blood cells (RBCs) from two independent assays with three technical replicates.

The *in vitro* antimalarial activities of the three *Anopheles* mosquito cecropin AMPs, An-cecA, An-cecB, and An-cecC, were tested. At the concentration of 50 µM, An-cecA had weak activity against *P. falciparum* 3D7 strain with an inhibition rate of only 18.7%. The inhibition rate of An-cecC was 40.16% and that of An-cecB reached 100% (Fig. 1a). Therefore, An-cecB was selected for further studies.

In order to explore the antimalarial activity of An-cecB, its active region was studied further. An-cecB was truncated into three fragments, An-cecB-1, An-cecB-2, and An-cecB-3, and their *in vitro* antimalarial activities against 3D7 were analyzed. Among the three truncated peptides, An-cecB-1 showed the strongest antimalarial activity. At 200 µM concentration, the maximum inhibition rate reached 83.67%, while the inhibition rates of An-cecB-2 and An-cecB-3 were 12.14% and 24.74%, respectively. This suggested that An-cecB-1 contains the main antimalarial active region of An-cecB (Fig. 1b). We then analyzed the *in vitro* IC50s of An-cecB and its truncated peptide An-cecB-1 against 3D7. An-cecB and An-cecB-1 inhibited 3D7 in a dose-dependent manner *in vitro*, and their IC50s were 12.4 and 68.63 µM, respectively (Fig. 1c).

To identify the antimalarial activity of An-cecB against drug-resistant *P. falciparum* strains, the IC50s of An-cecB against the artemisinin-resistant strain 803 and the chloroquine-resistant strain Dd2 were analyzed *in vitro*. An-cecB inhibited 803 and Dd2 in a dose-dependent manner with IC50s of 9.89 and Dd2 12.81 µM, respectively, which were not significantly different from its IC50 against 3D7 (Fig. 1d).

### The stage specificity of the antimalarial activity of An-cecB

**Fig 2:**
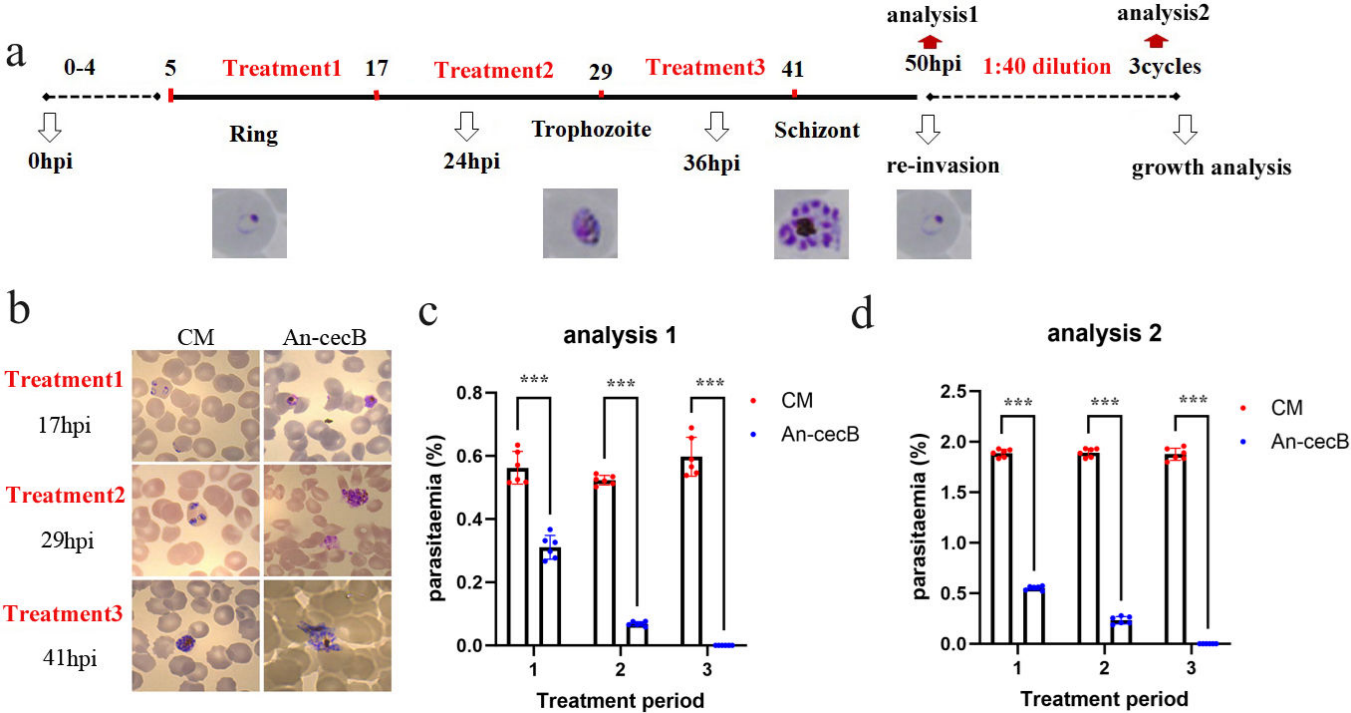
The antimalarial activity of An-cecB against different blood stages. (**a**) Schematic illustration of the experimental design. A representative image has been used to illustrate the parasite development stages. (**b**) The morphology of parasites at the end point of treatment1, treatment2, and treatment3 was evaluated by Giemsa’s solution-staining smears. (**c**) Parasitemia of reinvasion at 50 hpi after each treatment was measured by Giemsa’s solution-stained blood smears. (**d**) Parasites after each treatment were diluted by 1:40 after re-invasion at 50 hpi and cultured for 4 days while the third-cycle parasitemia was measured by Giemsa’s solution-stained blood smears. Data in c and d are individual replicates of 10,000 RBCs from two independent assays with three technical replicates. Statistical analyses between two groups were performed by Student’s *t*-tests, ***P < 0.001.

The activity of An-cecB against different blood stage parasites was evaluated. Parasites were exposed to An-cecB at a lethal concentration of 40 µM for 12 hours at the ring stage [5–7 hpi (hours post invasion)], trophozoite stage (17–29 hpi), and schizont stage (29–41 hpi) (Fig. 2a). Morphological analyses showed that An-cecB treatment significantly disrupted the morphology (Fig. 2b) and strongly blocked asexual development of parasites to the next cycle compared with control CM treatment at all stages (Fig. 2c). Parasites were then washed and cultured for 4 days followed by 1:40 dilution. The density of parasites in each drug treatment group was significantly lower than that of the control group treated with CM (Fig. 2d). All parasite stages were killed by An-cecB and did not reproduce in the next cycle (Fig. 2c and d), with this effect being particularly strong in the schizonts . Therefore, An-cecB was shown to be active against all stages of *Plasmodium* and showed the strongest activity against the schizonts.

### Cytotoxicity of An-cecB and the truncated peptides

**Fig 3:**
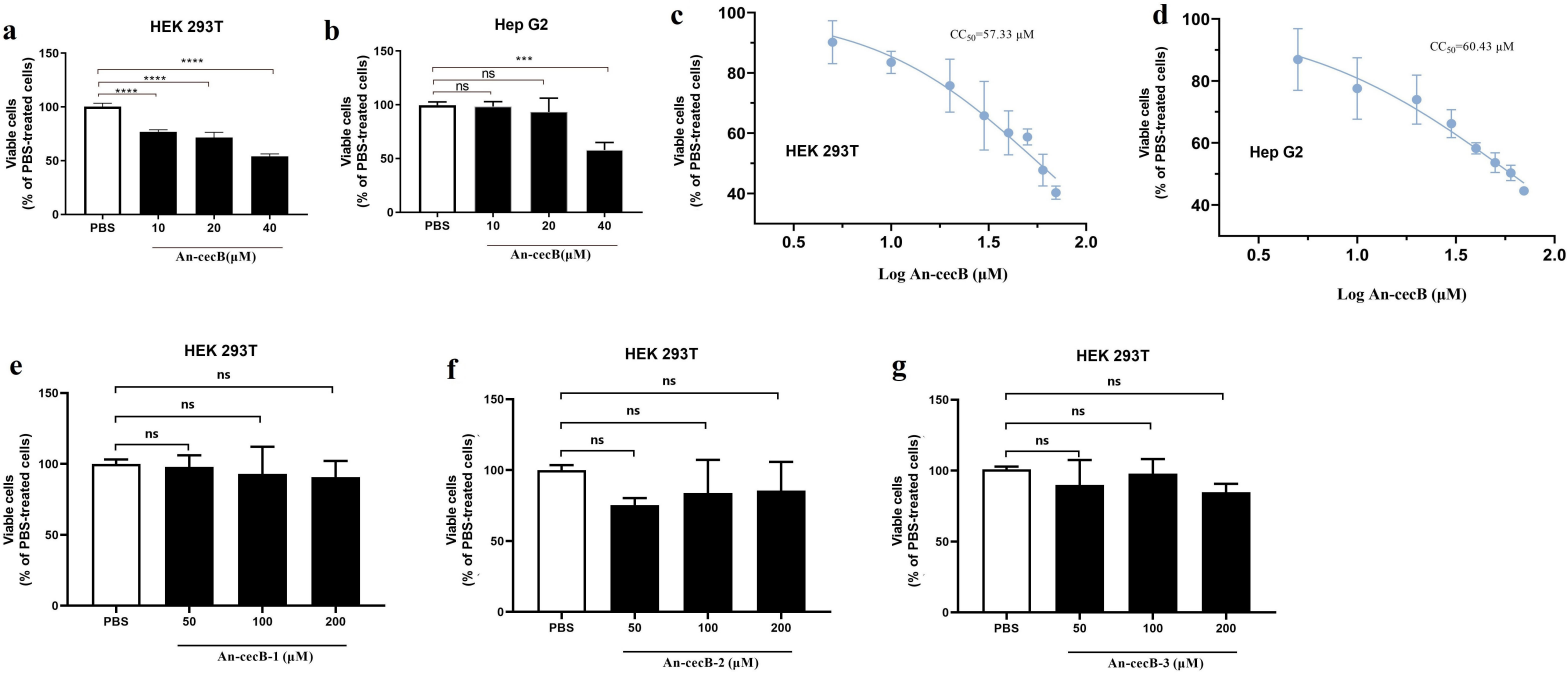
Cytotoxicity of An-cecB and its truncated peptides. Cytotoxicity of different concentrations of An-cecB to HEK 293T cells (**a**) and Hep G2 cells (**b**). CC50s of An-cecB to HEK 293T cells (**c**) and Hep G2 cells (**d**). No cytotoxicity of truncated peptides An-cecB-1 (e), An-cecB-2 (f), and An-cecB-3 (g) at different concentrations. Data are means ± SEM from two independent assays with three technical replicates. Statistical analyses between two groups were performed by Student’s *t*-tests,***P < 0.001; ns, not significant.

To determine the cytotoxicity of An-cecB to mammalian cells, the effects of An-cecB on HepG2 and HEK293T cells were measured. An-cecB dose-dependently inhibited the cell viability of HEK293T cells and was also toxic to HepG2 cells at a high concentration of 40 µM (Fig. 3a and b). An-cecB was toxic to HEK293T and HepG2 with CC50 values of 57.33 and 60.43 µM (Fig. 3c and d), resulting in selectivity indices (SI) of 4.62 and 4.87, respectively. The ideal drug would be one that either is not toxic or produces toxicity only at high concentrations but has the desired biological activity at very low concentrations, resulting in high SI values. Although the SI values of An-cecB were within the safe range under most criteria (>1) (31), significant cytotoxicity to mammalian cells was observed at high concentrations (Fig. 3a and b). Therefore, in order to explore the relationship between the cytotoxicity and antimalarial activity of the peptide and the region of cytotoxicity, three truncated peptides of An-cecB were assayed for their cytotoxicity to HEK293T cells. The three truncated peptides had no toxicity to HEK293T cells at various drug concentrations (Fig. 3e through g). It is interesting that although peptide An-cecB showed some cytotoxicity to mammalian cells, none of its three truncated peptides was cytotoxic, while An-cecB-1 retained most of its antimalarial activity.

### Antimicrobial activity

**Fig 4:**
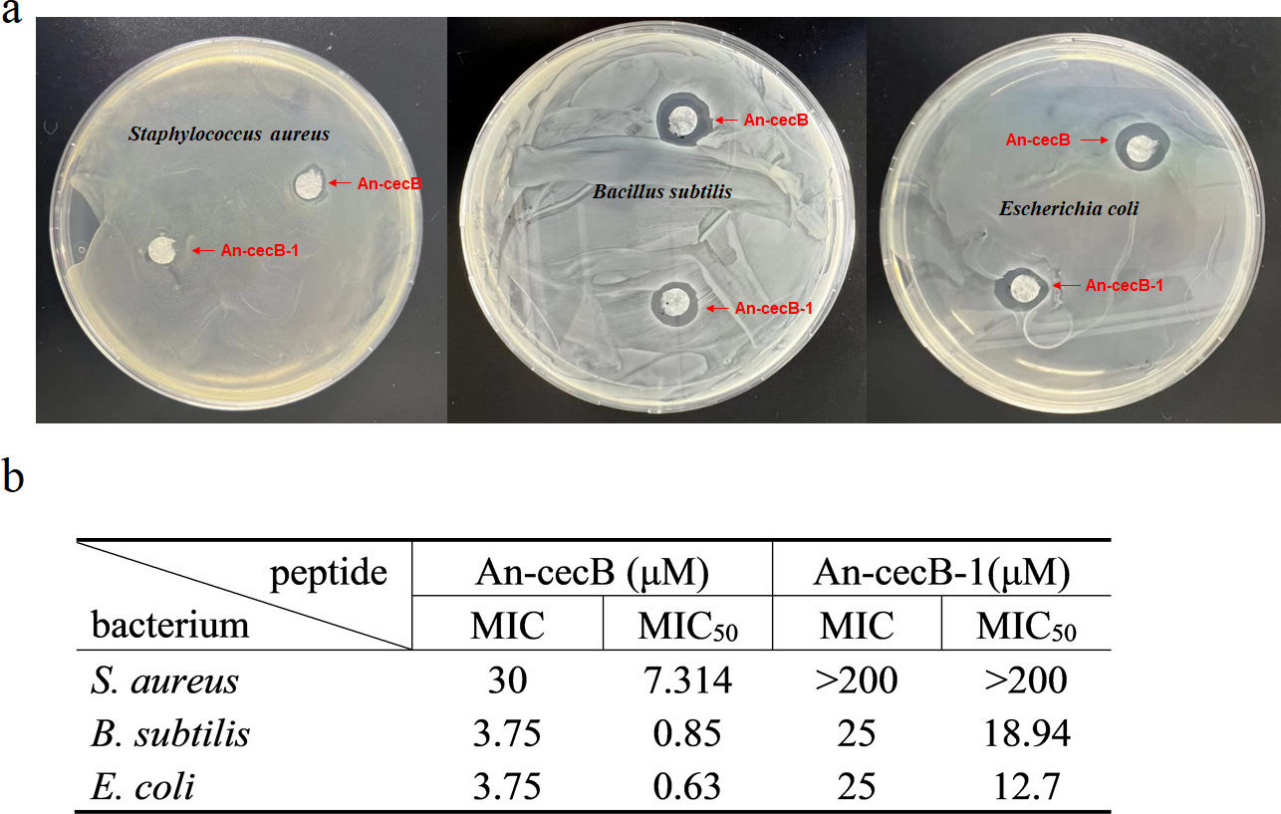
Antimicrobial activity of An-cecB and An-cecB-1. (**a**) Disc diffusion assay showing the antimicrobial activity of An-cecB and An-cecB-1 against *S. aureus*, *B. subtilis*, and *E. coli*. (**b**) Comparison of MIC and MIC50 of An-cecB and An-cecB-1 against *S. aureus*, *B. subtilis*, and *E. coli*. Data in b are means ± SEM from two independent assays with three technical replicates.

In addition to antimalarial activity, the antimicrobial activities of An-cecB and An-cecB-1 were analyzed. A disc diffusion assay showed that An-cecB had antibacterial activity against both Gram-positive and Gram-negative bacteria tested and this Head2antibacterial activity was significantly stronger than that of An-cecB-1 against all the tested bacteria (Fig. 4a). MIC and MIC50 also indicated that the antibacterial activities of An-cecB against *Staphylococcus aureus*, *Bacillus subtilis*, and *Escherichia coli* were stronger than that of An-cecB-1 (Fig. 4b).

### The antimalarial mechanism of An-cecB

**Fig 5:**
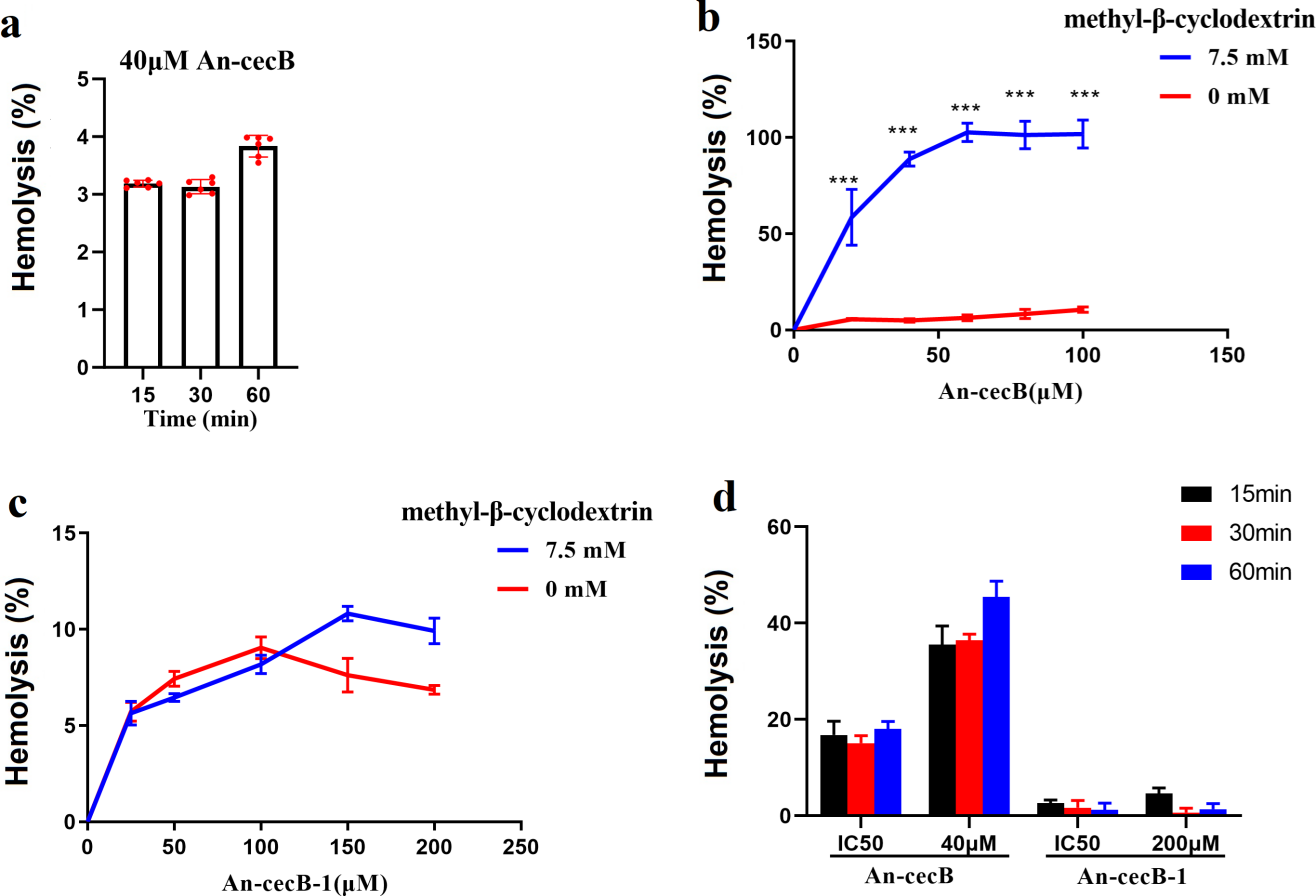
Hemolytic activity of An-cecB and An-cecB-1. (**a**) Hemolytic activities of 40 µM An-cecB on normal RBCs for different incubation times. Cholesterol depletion increases susceptibility of RBC membranes to An-cecB (**b**) but not to An- cecB-1 (c). (**d**) Hemolytic activity of An-cecB and An-cecB-1 to RBCs infected by schizonts. Data are individual replicates or means ± SEM from two independent assays with three technical replicates. Statistical analyses between methyl-β-cyclodextrin-treated and control groups were performed by Student’s *t*-tests, ***P < 0.001.

The main mechanism of action of AMPs is pore formation in cell membrane. In order to explore whether An-cecB could cause hemolysis of healthy erythrocytes while killing malaria parasites, its hemolytic activity on healthy erythrocytes was determined. The hemolysis rate of An-cecB to healthy erythrocytes was less than 2% after incubation for 60 min at the concentration of 10 and 20 µM (data not shown), and less than 5% even at the maximum concentration of 40 µM (Fig. 5a).

As the above hemolysis rates were measured with uninfected erythrocytes, we next sought to test the hemolysis rate of An-cecB against infected erythrocytes. To do this, we use methyl-β-cyclodextrin treatment to deplete cholesterol simulating the conditions in *Plasmodium*-infected erythrocytes. After cholesterol depletion, hemolysis of An-cecB-treated erythrocytes was significantly higher than that of controls (Fig. 5b). This result was consistent with the fact that An-cecB displayed its strongest activity against the schizont stages, which are the most cholesterol-depleting stages (32). Unlike An-cecB, the truncated peptide An-cecB-1 had no hemolysis effect on cholesterol-depleted erythrocytes (Fig. 5c). This may be due to the fact that the structural changes alter the physicochemical properties of peptides, leading to different antimalarial mechanisms. To confirm this, schizonts were collected and the hemolytic activities of An-cecB and An-cecB-1 to infected erythrocytes were also analyzed. We found that An-cecB specifically lysed infected erythrocytes, while An-cecB-1 did not (Fig. 5d).

**Fig 6:**
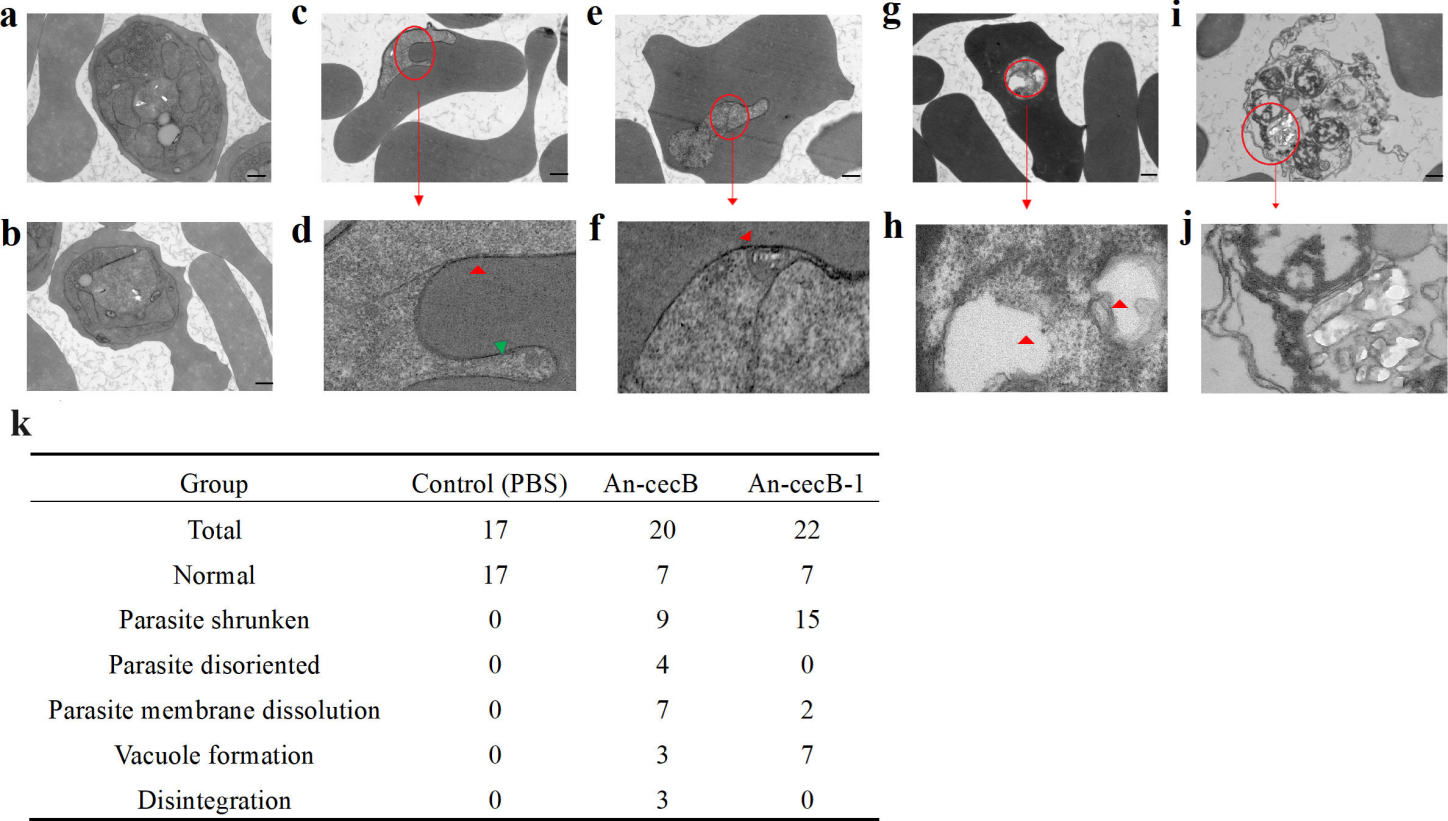
Transmission electron micrographs of *Plasmodium falciparum* after incubation with An-cecB. (**a, b**) Untreated control normal parasites; images are from different views of the same slide and may contain some overlap. Abnormal parasites (**c, e, g, and i**) treated by An-cecB or An-cecB-1 treated and their magnified art (**d, f, h, and j**). Treated group showed shrunken parasite bodies (**c, e, and g**), disoriented (green arrows in d) and dissolution parasite membranes (red arrows in d and f), vacuole formation (red arrows in h), and even parasite spill-over of erythrocytes and disintegration (**i, j**). (**k**) Statistics of the above abnormal morphologies in each group.

The direct, erythrocyte membrane-independent, effects of An-cecB and An-cecB- 1 on parasites were also evaluated. Transmission electron microscopy was used to analyze the changes in morphology and construction of parasites following exposure to the peptides. An-cecB induced disorientation (Fig. 6c) and dissolution (Fig. 6c through f) of parasite membrane and vacuole formation (Fig. 6g and h). Compared with normal parasites (Fig. 6a and b), An-cecB also caused shrinkage of the whole parasite body making the internal organelles difficult to discern (Fig. 6c , e and g), and parasite spill-over from erythrocytes and disintegration were observed (Fig. 6i and j). Therefore, An-cecB does not act only on infected erythrocytes but also directly affects parasites, disrupting their morphology and structure. Abnormal morphologies were quantified for each group (Fig. 6k) and show that in addition to causing parasite shrinkage, An-cecB had further effects on parasite membranes, including the orientation and integrity of the membrane, while An-cecB-1 was more likely to induce vacuole from inside the parasites.

### Assessment of antimalarial activity against *Plasmodium berghei* in mice

**Fig 7:**
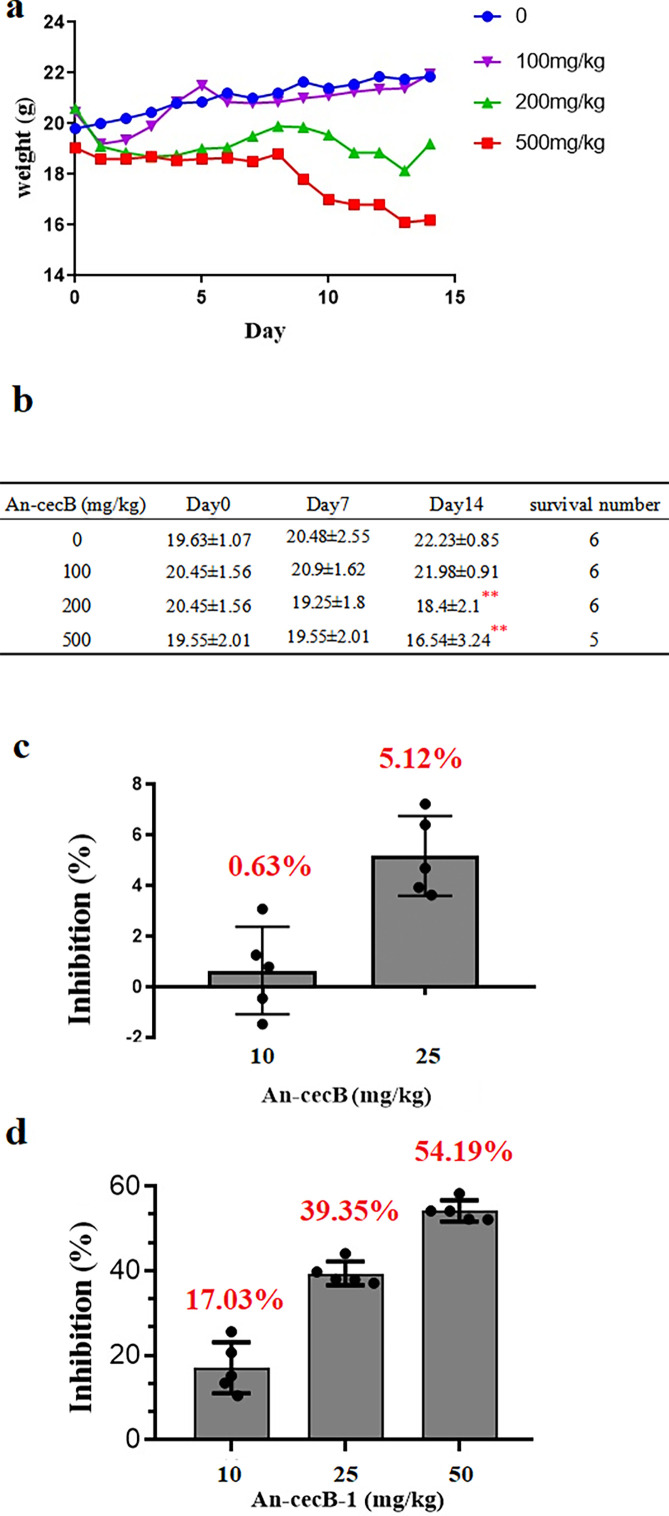
Evaluation of *in vivo* antimalarial effects of peptides. (**a**) Effect of An-cecB acute oral toxicity test on body weight of mice. (**b**) Body weight on days 0, 7, and 14 and survival rates of mice in acute oral toxicity test with An-cecB at doses of 0, 100, 200, and 500 mg/kg (*n* = 6). (**c**) Inhibitory activity of An-cecB at doses of 10 and 25 mg/kg against *Plasmodium berghei in vivo*. (**d**) Inhibitory activity of different doses of An-cecB-1 against *P. berghei in vivo*. Data are means ± SEM from two independent assays with six mice each time. Statistical analyses between two groups were done by Student’s *t*-tests, **P < 0.01.

The *in vivo* antimalarial activity of An-cecB was evaluated using a 4-day suppressive test in *Plasmodium berghei*-infected mice. Prior to testing for antimalarial activity *in vivo*, an acute toxicity test of An-cecB was performed. The body weight of mice was continuously monitored and recorded for 14 days (Fig. 7a). At an An-cecB dose of 500 mg/kg, one mouse died on the Day8, while there were no deaths observed at 100 and 200 mg/kg dosages (Fig. 7b). The body weight of mice treated with 200 and 500 mg/kg decreased significantly compared with the control group (*P <* 0.01) on Day14 (Fig. 7b).

The 4-day suppressive test showed that at the concentrations of 25 mg/kg, An-cecB inhibited the growth of *P. berghei* by only 5.12%, whereas at concentrations up to 50 mg/kg, all infected mice died before the end of the experiment, 10 mg/kg chloroquine was used as the positive control (Fig. 7c).

Since An-cecB-1 appears to have a different antimalarial mechanism, its *in vivo* antimalarial activity was also tested. Interestingly, although the antimalarial activity of An-cecB-1 was not as effective as An-cecB *in vitro*, it was much stronger than that of An-cecB *in vivo*. The results showed that An-cecB-1 inhibited the growth of *P. berghei* in a dose-dependent manner. At the doses of 10, 25, and 50 mg/kg, the inhibition rates of An-cecB-1 to *P. berghei* were 17.03%, 39.35%, and 54.19%, respectively (Fig. 7d).

## DISCUSSION

Current control methods for malaria are failing, mostly due to the emergence and spread of mutant parasites resistant to the commonly used antimalarial drugs (33, 34). New drugs with novel antiparasitic modes of action are required. Given this, AMPs whose modes of action involve perforation of cell membranes may offer interesting solutions. Indeed, many broad-spectrum AMPs from different sources show varying degrees of antimalarial action. Such as ankyrin peptide (AnkP), which can be delivered to parasite-infected RBCs, NK-2 can rapidly internalize parasite-infected RBCs and affect intracellular parasite viability with little impact on healthy RBCs (33). As eukaryotes, *Plasmodia* may be probably incompatible with the general notion that selective AMP preferentially targets negatively charged prokaryote membranes. However, the phospholipid composition of membranes from intraerythrocytic parasites is significantly different from that of their host RBCs, and, due to *Plasmodia* parasitism, the composition of the host RBC membranes is also different from that of healthy RBCs, which may explain that AMP exhibits antimalarial activity through membrane mechanisms (33). Importantly, such a mode of action would make these compounds less prone to the evolution of drug resistance than traditional drugs.

We have shown here that An-cecB, an AMP from *Anopheles* mosquitoes, is able to inhibit the growth of both drug-resistant and sensitive strains of *P. falciparum* development. Hemolysis assays showed that An-cecB had minimal hemolytic activity on normal erythrocytes but did lyse erythrocytes infected with *P. falciparum* parasites and those treated with methyl-β-cyclodextrin. The results show that An-cecB can specifically target the membranes of infected erythrocytes, a promising property when developing in the development of new antimalarial drugs. Besides its hemolytic activity against *P. falciparum*-infected erythrocytes, transmission electron microscope analysis showed that An-cecB seriously affected the morphology and structure of *P. falciparum*, indicating that it not only had an effect on infected erythrocytes but also had a direct effect on the parasites themselves.

*Anopheles* mosquitoes have developed complex immune systems to defend themselves from invading pathogens, and AMPs are an important part of their defense systems. We have shown that three cecropins from *Anopheles arabiensis* all exhibited antimalarial activity *in vitro*, with the cecropin B genes showing the strongest activity. In addition to cecropin, there are many other peptides involved in the mosquito defense system. To overcome the burden of mosquito-borne diseases, multiple control strategies are needed. Population replacement with genetically modified mosquitoes carrying an anti-pathogen effector gene has been suggested as one such strategy (35). Genetic engineering of malaria-resistant transgenic mosquitoes has been achieved through the induction of expression of exogenous AMPs from other insects (36) or through the overexpression of native AMPs (35).

An-cecB was shown to be cytotoxic to mammalian cells. However, it seems likely that this peptide would not be toxic to mosquito cells, as it is naturally produced by the mosquitoes themselves. The *in vitro* antimalarial activity of An-cecB raises the possibility of using it as an antimalarial within mosquitoes through induction of its overexpression, although this remains to be tested. One caveat to this approach is that overexpression of a protein with known antibacterial properties may affect the mosquito immune system in complex ways through disruption of the insects’ natural microbiome.

Although An-cecB displayed minimal hemolytic activity against normal erythrocytes, it was shown to be toxic to mammalian cells. We truncated An-cecB into the three peptides An-cecB-1, An-cecB-2, and An-cecB-3. An-cecB-1 retained most of the antimalarial activity but not the cytotoxicity of An-cecB. Although the antimalarial activity of An-cecB-1 was slightly inferior to An-cecB *in vitro*, its antimalarial activity *in vivo* was far better than that of An-cecB. This may be due to structural changes in the peptides leading to changes in their physicochemical properties and perhaps modes of action. This hypothesis was supported by the fact that An-cecB-1 had minimal hemolytic activity to erythrocytes treated by methyl-β-cyclodextrin or those infected by the *P. falciparum* parasite. In addition to its hemolytic activity, transmission electron microscope analysis showed that An-cecB has further effects on the membrane of parasite, including the orientation and integrity of the membrane, while An-cecB-1 was more likely to result in vacuole formation within parasites.

An-cecB had stronger antimalarial activity *in vitro* but was much weaker *in vivo* than that of An-cecB-1. It is possible that in addition to direct parasite killing, An-cecB-1 may also participate in immune regulation against parasites *in vivo*.

Through modification of the original peptide, we were able to produce a compound, An-cecB-1, with reduced cytotoxicity and improved antimalarial activity, through the simple strategy of truncating the original peptide into three fragments. Given this success, we hope that further modification and refinement may lead to more effective and safer peptides.

In conclusion, the *Anopheles* mosquito-derived AMP An-cecB possesses antimalarial activity as well as cytotoxicity, while its truncated peptide An-cecB-1 retained most of its antimalarial activity without the cytotoxicity and displayed better antimalarial activity *in vivo*. The antimalarial activity *in vitro* suggests that it is highly likely to have antimalarial activity in mosquitoes as well, but this remains to be verified. As peptides are highly modifiable, they can be refined to enhance activity and avoid toxicity. Because they act in a different way than traditional small-molecule drugs, AMPs are a potential tool to address the problem of drug resistance in malaria parasites, and we contend that AMPs in *Anopheles* mosquitoes constitute a promising resource for the development of new antimalarial drugs.

## MATERIALS AND METHODS

### Animals

Female BALB/c mice (SPF grade) were purchased from SiPeiFu (Beijing) Biotechnology Co., Ltd. All animals were housed in a pathogen-free facility in accordance with the Guide for the Care and Use of Medical Laboratory Animals (Ministry of Health, People’s Republic of China, 1998).

### Peptide selection, design, and synthesis

The peptides in this study are three cecropins of *Anopheles arabiensis* from the NCBI database, with the accession numbers XP_040173531 (An-cecA), XP_040173530 (An-cecB), and XP_040172706 (An-cecC). The peptides were synthesized with the signal peptides removed and ending with the predicted terminal amino acid of the cecropin region. Considering the number of amino acids, the number of charges, and the position in the original peptide, the peptide was bisected to obtain An-cecB-1 and An-cecB-2, and the middle part of the original peptide was truncated to form An-cecB-3. The truncated three peptides contain the anterior, posterior, and middle parts of the peptide, respectively. For enhancing the stability and bioactivity of the peptides, each peptide was synthesized with C-terminus amidation. All peptides were synthesized by GL Biochem (Shanghai) Ltd. (Shanghai, China). The synthesized peptides were analyzed by reversed-phase high performance liquid chromatography and mass spectrometry to confirm purity higher than 98%. All the peptides are hydrosoluble and were dissolved in PBS to make 20 mM stock solution.

### Parasite strains, cells, and bacterial strains

All the parasite strains used in the study, including *P. falciparum* 3D7 (sensitive strain), *P. falciparum* 803 (artemisinin-resistant strain), *P. falciparum* Dd2 (chloroquine-resistant strain), and *P. berghei* (ANKA), were stored in our laboratory. HepG2 and HEK293T were the preserved cells from our laboratory. Human erythrocytes were from Wuxi Blood Center. The bacterial strains including Gram-positive bacterium *Staphylococcus aureus* (ATCC 6538), *Bacillus subtilis* (ATCC), and Gram-negative bacteria *Escherichia coli* (ATCC 25922) were purchased from Guangdong Microbial Culture Collection Center.

### Plasmodium culture

Blood stages of the laboratory clones *P. falciparum* 3D7 (sensitive strain), *P. falciparum* 803 (artemisinin-resistant strain), and *P. falciparum* Dd2 (chloroquine-resistant strain) were cultured *in vitro* according to the method of Trager and Jensens with minor modifications (37). Briefly, cultures were maintained in O-positive human erythrocytes (RBCs) as host cells at 2% hematocrit and maintained in complete medium (CM) [RPMI-1640 culture medium supplemented with NaHCO3 (2 mg/mL), hypoxanthine (50 µg/mL), HEPES (5.96 mg/mL), albumax II (1%), and gentamicin (40 µg/mL)]. The cultures were diluted to 1% parasitemia and 2% hematocrit in CM and incubated in a humidified atmosphere with 5% O2, 5% CO2, and 90% N2 at 37°C. Prior to experiments, the cultures were highly synchronized for ring stages using 5% sorbitol twice at a 40-h interval. Briefly, ring stage cultures were centrifuged and the pellet was washed once in incomplete medium (ICM, RPMI-1640 without addition). Then, the pellet was resuspended in 5% sorbitol and incubated at 37°C for 10 min. After incubation, the suspension was centrifuged and the pellet was washed once and resuspended in medium. The development of the parasite was observed using light microscopy, and parasitemia was determined by counting at least 10,000 RBCs on Giemsa’s solution-stained thin blood smears.

### Assessment of *in vitro* antimalarial activity of peptides

Highly synchronous ring stage parasites were used to investigate the *in vitro* antimalarial activity of the peptides. For preliminary screening of *in vitro* antimalarial activity, a 3-day inhibition assay was used to test the antimalarial activity of An-cecA, An-cecB, and An-cecC. Briefly, after highly synchronization, an aliquot of parasite inoculum (200 µL) with 1% parasitemia and 2% hematocrit was added into each well of a 96-well plate followed by An-cecA, An-cecB, or An-cecC peptide (dissolved in PBS and diluted with RPMI 1640) at final concentrations of 50 µM. For IC50 assay, a series of concentrations of peptides were added to the parasite culture. The concentrations of An-cecB were 5, 15, 20, 25, 30, 35, and 40 µM, while those of An-cecB-1 were 20, 30, 40, 50, 60, 70, 80, and 90 µM. After 72 hours of incubation, the antimalarial effects of peptides were estimated. Thin blood films stained with Giemsa’s solution were counted under a microscope. The parasitemia of cultures treated with peptides was compared with that of control. IC50 was calculated by GraphPad Prism. Two independent assays with three technical replicates each time were conducted.

### Hemolytic activity

Hemolysis assays were performed using human RBCs according to previous reports (38). Briefly, serial dilutions of peptides were incubated with 2% hematocrit RBCs (in PBS) at 37°C for three different time points (15, 30, and 60 min) in triplicates. After incubation, the RBCs were centrifuged for 10 min at 1,000 × *g* and the absorbance of the supernatants at 540 nm was read by a spectrophotometer to assess the level of the released hemoglobin. Maximum hemolysis was determined by substituting the sample with 1% Triton X-100, which completely lyses the cells. PBS showed no hemolytic activity and was used as a negative control. Hemolysis of peptides was calculated using the following formula: Hemolysis (%) = (A540 of peptide- A540 of PBS) * 100/A540 of Triton X-100. Two independent tests with three technical replicates were performed for each hemolysis assay.

Cholesterol is the main component of the RBC membrane and is important for maintaining its stability (39). *Plasmodium falciparum* infection efficiently depletes the red blood cells of cholesterol, which renders parasite membranes susceptible to lysis (40). Therefore, methyl-β-cyclodextrin treatment was used to simulate the RBC cholesterol depletion caused by infection with *Plasmodium.* RBCs were washed once and then resuspended with PBS at 0.5% hematocrit. After treatment with the indicated concentration (7.5 mM) of methyl-β-cyclodextrin (Sigma) in RPMI for 2 hours at 37°C, the treated RBCs were washed with PBS and hemolysis assays were performed as described above. Two independent tests with five technical replicates were performed for each hemolysis assay.

To test the hemolytic activity of the peptides against RBCs infected by *Plasmodium*, schizonts of the *P. falciparum* 3D7 strain were collected. The parasite culture of mainly schizonts was centrifuged and resuspended to 50% hematocrit. Then, the resuspended culture was carefully laid on top of the gradient with 3 mL 70% and 3 mL 40% percoll-sorbitol solution. After centrifugation at 2,300 × *g* for 20 min at 20°C, schizonts from the 40/70% interface were collected and washed twice with 10× volume of ICM. The collected parasites were resuspended in PBS to 2% hematocrit for hemolysis assay as described above. Two independent tests with three technical replicates were performed for each hemolysis assay.

### Cytotoxicity

The mammalian cell lines HepG2 and HEK293T were used for cytotoxicity tests. The cells were cultured in Dulbecco’s modified Eagle medium supplemented with 10% fetal bovine serum and 1% penicillin/streptomycin in an incubator at 37°C with 5% CO2. Cells were seeded at a density of 1 × 105 cells/mL per well into a 96-well plate overnight and treated with different concentrations of peptides for 48 hours. To analyze the CC50 of peptides, a series of concentrations of peptides (0, 5, 10, 20, 30, 40, 50, 60, and 70 µM) were set to detect their cytotoxic concentrations. Then, 10% CCK-8 solution was added into each well and incubated for another 2 hours. The absorbance was monitored by a microplate reader at 450 nm. PBS was used as a negative control and the inhibition of each drug was normalized to that of the PBS control. CC50 was calculated by GraphPad Prism. Two independent assays with three technical replicates each time were conducted.

### Stage-specific parasite inhibition assay

To analyze the stage-specific parasite inhibition, highly synchronized ring-stage parasites of the 3D7 strain were cultured in 48-well plates with a starting parasitemia of 0.5% ~ 1% at a hematocrit of 2%. The tested peptide at a concentration of 40 µM was added to ring stage parasites (5 hpi), trophozoites (17 hpi), and schizonts (29 hpi). After treatment for 12 hours, the parasites were washed three times to remove the peptide and the re-invaded parasites (50 hpi) were analyzed. The parasitemia (50hpi) was diluted 1:40 by new RBCs and cultured for a further 4 days. CM was used as a blank control. Parasitemia was counted under a microscope. Two independent assays with three technical replicates each time were performed.

### Antimicrobial assays

Gram-positive bacteria *S. aureus*, *B. subtilis*, and Gram-negative *E. coli* were used in antimicrobial assays. Bacteria were first grown in LB (Luria-Bertani) broth to an absorbance of 0.8 at 600 nm. The antibacterial behavior of the peptides was studied *via* the agar disc diffusion assay and broth microdilution method (41, 42). The agar plates were made by pouring 20 mL of LB broth containing 1.5% agar into 60-mm Petri dishes. Then, a 100-µL bacterial suspension (106 CFU/mL) was spread over the LB agar plate. Ten microliters of the 2-mg/mL peptide sample was carefully dropped onto sterile 2-mm diameter paper discs that had been previously attached to the plate. The plates were carefully placed in an incubator at 37°C for 24 hours. If an examined sample had antimicrobial activity, a clear zone was observed around the filter paper representing inhibition of bacterial growth. The MIC was determined by the broth dilution method, and the absorbance value was measured at an optical density at 600 nm (OD600) to determine the inhibition rate of peptides at different concentrations against each bacteria. Then, the MIC at which no visible growth occurred was recorded (38). The MIC50 was calculated by GraphPad Prism. Two independent assays with three technical replicates each time were performed.

### Transmission electron microscope analysis

3D7 strain parasites with parasitemia of 3% ~ 5% at a hematocrit of 2% were planted in 12-well plates, 3 mL/well. A 40-µM concentration of An-cecB or 200 µM An-cecB-1 was added and incubated for 30 min or 60 min. PBS was used as a control.

After incubation, the parasites were washed three times with ICM, and the electron microscope fixative solution (provided by the company) was added to keep the parasites clumped for 2 hours at room temperature. Finally, the samples were sent to a commercial company at 4°C for transmission electron microscope analysis.

### Acute toxicity test

Mice were housed under standard conditions at a temperature of 24 ± 2°C and a humidity of 50%–80%. Lighting was alternated between day and night every 12 hours. All mouse groups consisted of six animals per cage, and these were fed *ad libitum* with SPF-grade stock diet and water. All the experimental mice were adapted to the animal room for 1 to 2 weeks before the experiment.

For the acute toxicity test, the mice were divided into four groups. Each group contained six mice. Mice were fasted for 2 hours before feeding with a single oral dose of peptide. The mice in each group were fed with peptide at a single oral dose of 500, 200, and 100 mg/kg body weight. Normal saline (1 mL) was used as control. The general behavior of each mouse was observed continuously for the first 1 hour, then intermittently every 4 hours, and thereafter over a period of 24 hours (43). The mice were observed for up to 14 days, and the body weight changes were recorded.

### Four-day suppressive test

The *in vivo* antimalarial activity of peptides was evaluated using a 4-day suppressive test in *P. berghei*-infected mouse model mice (44). The mice were divided into five groups of six mice. Donor mice were infected with 200 µL of *P. berghei* parasite inoculum by intraperitoneal injection. Parasitized blood was collected from the tail vein of donor mice and diluted with 0.9% sodium chloride solution. Mice were then infected with saline suspension of 1 × 107 parasitized erythrocytes (0.2 mL) by intraperitoneal injection (Day0). Four hours after infection, each of the three groups of mice was treated with peptide by intraperitoneal injection at daily doses of 10, 25, and 50 mg/kg for 4 consecutive days. Positive control mice were treated with chloroquine at daily doses of 10 mg/kg and negative control mice with normal saline. On day 4 (96 hours post infection), the parasitemia of each mouse was determined under a light microscope by examination of Giemsa’s solution-stained thin blood smears prepared from mouse tail blood (28). The mean parasitemia in each group of mice was used to calculate the % suppression for each dose using the following formula:

%suppression = (parasitemia of negative control − parasitemia of test) * 100/parasitemia of negative control.

The antimalarial activity of the peptides was determined from the ratio of the percentage of parasite reduction in treated and negative control groups (44). Results were expressed as median (range) values.
